# Type I Jejunal Atresia in Identical Twins: A Rare Occurrence

**DOI:** 10.21699/jns.v6i3.616

**Published:** 2017-08-10

**Authors:** Amrollah Salimi, Shervin Rashidi Nia, Seyed Shahin Eftekhari, Mahsa Besharati, Sara Shahmoradi

**Affiliations:** 1 Department of Pediatric Surgery of Hazrat Masoume Hospital, Qom University of Medical Sciences, Qom, Iran; 2 Student Research Committee, Qom University of Medical Sciences, Qom, Iran

**Keywords:** Jejunal atresia, Twin, Genetic inheritance

## Abstract

Jejunoileal atresia is of familial and non-familial in origins and classified into four different types. We herein report a rare occurrence of type I jejunal atresia in identical twins who were presented with neonatal intestinal obstruction. This report points towards common etiology of atresia in our cases and factors more than vascular accident appear to be involved.

## INTRODUCTION

Jejunoileal atresia (JIA) is a leading cause of intestinal obstruction in neonates [1]. The incidence rate of this complication is approximately 1 in 5000 live births [2]. Various types of jejunal atresia were defined as the Grosfeld’s classification system [3]. Intra-uterine vascular accidents, either primary or secondary, are thought to be the underlying etiology [2], though another classification is familial and non-familial types. In this report, we describe a rare occurrence of type I jejunal atresia (non-familial) in identical twins.

## CASE REPORT

Identical female twins were born through cesarean delivery at 39 weeks, with the birth weight of 2630 and 2840 g, respectively. A history of polyhydramnios was noted during pregnancy. Both neonates developed intestinal obstruction. Abdominal radiographs showed a triple bubble sign. A pre-operative diagnosis of jejunal atresia was formed for both. At surgery on 2nd day of life, type I jejunal atresia was found in both neonates (Fig.1,2) with no evidence of any other anomalies. Jejuno-jejunal anastomoses were performed after resecting atretic portions in both twin. Postoperative recovery remained uneventful.

**Figure:**
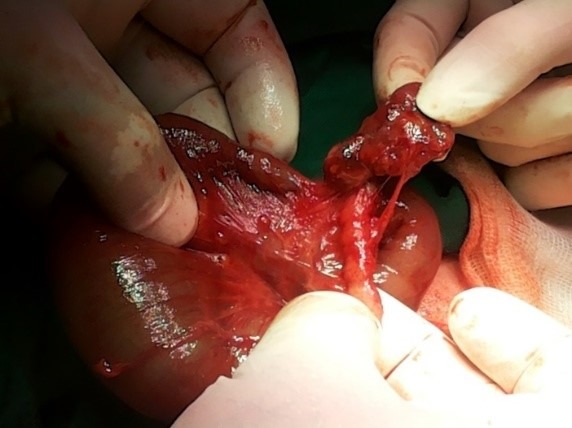
Figure 1. Intra-operative photograph confirming a type I jejunal atresia.

**Figure:**
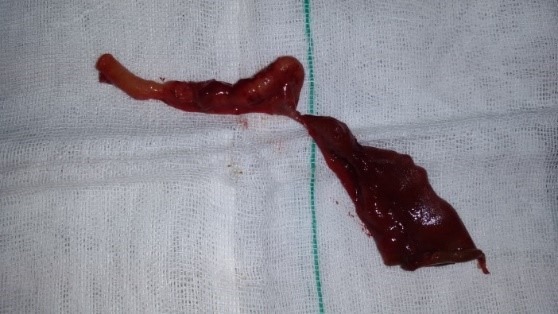
Figure 2. Atretic portion of the jejunum after resection.

## DISCUSSION

Intra-uterine ischemic insult is believed to be the underlying cause of jejunal atresia [2]. Familial inheritance and genetic susceptibility are supported in types IIIb and IV jejuno-ileal atresia [4]. Jejunal atresia was reported in 3 siblings of a family suggestive of its possible inheritance through an autosomal recessive gene [5]; Nevertheless, the authors did not describe the type of the atresia in their cases. Shorter et al. [6] reported a case of type IIIb jejunal atresia in a boy with history of jejunal atresia in another sibling. Olson et al [7] reported type IV jejunal atresia in identical twins supporting the genetic inheritance of this type of jejunal atresia. We reported two cases of type I jejunal atresia in identical twins. To the best of our knowledge, this is the first report of this type of jejunal atresia in identical twins. Contribution of genetic inheritance in developing this condition can be speculated in the index cases.

## Footnotes

**Source of Support:** None

**Conflict of Interest:** None
